# Improving the capacity of community-based workers in Australia to provide initial assistance to Iraqi refugees with mental health problems: an uncontrolled evaluation of a Mental Health Literacy Course

**DOI:** 10.1186/s13033-018-0180-8

**Published:** 2018-01-15

**Authors:** Maria Gabriela Uribe Guajardo, Shameran Slewa-Younan, Betty Ann Kitchener, Haider Mannan, Yaser Mohammad, Anthony Francis Jorm

**Affiliations:** 1 Mental Health, Translational Health Research Institute, School of Medicine, Western Sydney University, Sydney, Australia; 2 Centre for Mental Health, Melbourne School of Population and Global Health, University of Melbourne, Melbourne, Australia; 3 Mental Health First Aid Australia, Melbourne, Australia; 4 Translational Health Research Institute, School of Medicine, Western Sydney University, Sydney, Australia; 5 Bankstown Community Mental Health Services, Sydney, Australia; 6 Ware St Medical & Dental Centre, Sydney, Australia

**Keywords:** Mental Health Literacy Course, Community-based workers, Iraqi refugees, Mental health problems

## Abstract

**Background:**

Australia is a multicultural nation with a humanitarian program that welcomes a large number of Iraqi refugees. Despite the high prevalence of trauma related disorders, professional help-seeking in this group is very low. This study sought to evaluate a face-to-face mental health literacy (MHL) Course that teaches community-based workers how to provide initial help to Iraqi refugees with depression and post-traumatic stress disorder (PTSD) related problems.

**Methods:**

An uncontrolled pre, post and follow-up design was used to measure improvement in MHL in community-based workers assisting Iraqi refugees.

**Results:**

Eighty-six participants completed the pre- and post-training questionnaires. Forty-five (52%) completed all 3-time point questionnaires. Fifty-six percent (48/86) of participants were able to correctly recognise ‘PTSD’ as the problem depicted in a vignette before the training. This increased to 77% (66/86) after training and was maintained at follow-up with 82% (37/45) correctly recognising the problem *(p* = 0.032*)*. Recognition of depression also increased from 69% (59/86) at pre-training to 83% (71/86) after training and to 82% (37/45) at follow-up. There was a significant increase in perceived helpfulness of professional treatments for depression after training (*p* < 0*.001 at post*-*training, p* = 0.010 *at follow*-*up*). Significant changes were reported in confidence of participants when helping an Iraqi refugee with PSTD (*p* < 0.001 *at post*-*training, p* < 0.001 *at follow*-*up*) and depression *(p* < 0.001 *at post*-*training, p* = 0.003 *at follow*-*up)*. A decrease were also found on social distance mean scores associated with PTSD *(p* = 0.006 *at post*-*training, p* < 0.001 *at follow*-*up*) and depression (*p* = 0.007 *at follow*-*up*). Changes were not significant following training for offering help and helping behaviours in both PSTD and depression vignettes and, the ‘dangerous/unpredictable’ subscale in the depression vignette.

**Conclusion:**

This training is a recommendable way to improve and better equip staff on how to respond to mental health crises and offer Mental Health First Aid in a culturally sensitive manner to Iraqi refugees.

**Electronic supplementary material:**

The online version of this article (10.1186/s13033-018-0180-8) contains supplementary material, which is available to authorized users.

## Background

### Australian refugee resettlement

Australia is a multicultural nation with a humanitarian program that seeks to welcome refugees fleeing violence and oppression in their country of origin [1]. One of the top source countries for refugee application to Australia is Iraq [2] with almost 14,000 Iraqi refugees resettled in Australia in the period 2010–2015 alone [3]. Recently, Australia committed to resettle additional places for refugees from Iraq and Syria in response to the sectarian violence from extremist groups in the Middle East [4].

Under the Australian Humanitarian Programme, there are services that provide both immediate and ongoing assistance designed to facilitate the long-term settlement of new arrivals into Australian society [5]. These programs include assistance in finding employment, accommodation, improving English skills, and providing health care, all with the common goal of building individuals’ self-reliance and fostering connections with mainstream services [5]. In Australia, state government health organisations are responsible for the assessment of refugee health status in the areas of immunisation, oral health, infectious diseases, reproductive health and mental health, amongst others [6]. Additionally, resettlement social services are commonly offered by non-government organisations. In New South Wales (NSW), and specifically in the South Western Sydney area, there are several organisations, such as migrant resources centres, ethnic community-based organisations and English colleges, which are responsible for helping refugees to settle [7]. The concentration of such organisations in the South Western Sydney area reflects the high density of resettled refugees residing locally, being one of the most culturally-diverse districts nationwide [8]. In terms of those who provide these social services, their training is often diverse (English tutors, community case workers) [7], but most often lacks a component of mental health training. Given that these workers are often the first point of contact and play an important role in assisting resettling refugees to help navigate the Australian system [7], having basic mental health skills would be valuable.

### Refugee mental health

It is well known that refugees are at high risk of poor mental health outcomes due to pre-displacement events such as high level of trauma exposure, violence and deprivation suffered in their country of origin along with the challenges faced during and after the resettlement period [9, 10]. The literature highlights the particular nature of the refugee context as significant determinants of poor mental health for refugees. This in turn leads to issues of greater complexity of care and reduced health outcomes [11, 12], and Iraqi refugees are not an exemption [13]. An Australian-based study [14] conducted with resettled Iraqi refugees that aimed to explore psychological status and mental health literacy reported that over 30% of participants met the threshold for clinically significant posttraumatic stress disorder (PTSD) symptomatology and almost 40% of them experienced severe psychological distress which greatly exceeds rates reported for the Australian general population [15].

### Help-seeking in refugees: cross-cultural factors

Despite the greater need for specialised mental health care, help-seeking amongst Iraqi refugees is reportedly low. In an Australian-based study conducted to explore help-seeking and psychological trauma in Iraqi refugees, it was reported that only 19% of the sample sought any kind of help (professional and non-professional) and of those, less than 45% reported seeking professional help from a general practitioner, psychiatrist or psychologist with the majority reporting help-seeking from a family member [16].

There are, of course, multiple factors impacting on professional help-seeking by refugees with mental health problems. One of the most significant barriers to professional help-seeking not only for Iraqi refugees but also for the general public, is the stigma attached to mental health problems [17, 18]. Perceived and personal stigma, discrimination and social distance towards people with mental health problems are often reported and have been well researched for some decades now [17, 18]. Within the Iraqi community, it is common for men not to seek help for fear of appearing weak when they are expected to be strong and support their families [19]. This is strongly linked with the discrimination that many refugees face if someone from their community knows about their mental health problems [19, 20].

Other barriers to professional help-seeking can include unaffordable transportation and low English proficiency [21]. More broadly, there is a lack of experience and understanding on how to navigate the Western healthcare system [22]. It has been proposed that refugees might tend to seek informal help due to the lack of formal mental health assistance available for the public in their country of origin [22]. In addition, It has been reported that less acculturated refugees would be less likely to discuss their problems and less likely to believe in usefulness of professional help than those who were more acculturated [23].

### A way forward: improving mental health literacy of resettlement workers

According to the World Health Organisation (WHO), in an optimal mental health service framework the role of informal service provision is essential, with a recommendation to upskill local community members such as lay volunteers, community workers, humanitarian aid workers, and other professionals such as teachers and police officials [24]. Functions performed by these informal service providers can include assistance with activities of daily living and reintegration into the community, prevention and promotion services, practical support, crisis support and identification of mental health problems, and referral to health services. Some of the WHO initiatives that have been developed in partnership with other organisations included training Kwazakhele volunteers and community members to improve their mental health literacy, improving support of and outreach to individuals who have dropped out from formal services in the in the Eastern Cape Province of South Africa [24].

Given the lack of mental health training in many people who assist refugees, and the high prevalence of mental disorders reported in refugees who are unlikely to seek professional help, the current study aimed to provide initial training to staff working in resettlement organisations and community-based settings. Specifically, this study sought to improve metal health literacy levels by delivering a Mental Health Literacy Course to those who work in the resettlement of refugees, with the aim to better equip these workers to respond to mental health crises and offer first aid to this group. The term ‘mental health literacy’ refers to, ‘knowledge and beliefs about mental disorders which aid their recognition, management or prevention’ [25]. This encompasses (a) the public’s knowledge of how to prevent mental disorders, (b) recognition of when a disorder is developing, (c) knowledge of help-seeking options and treatments available, (d) knowledge of effective self-help strategies for milder problems, and (e) first aid skills to support others affected by mental health problems [25]. Increasing mental health literacy can achieve an important goal of empowering the public with an understanding of mental disorders, thereby facilitating prevention, early intervention and treatment within the community [26].

**Fig. 1:**
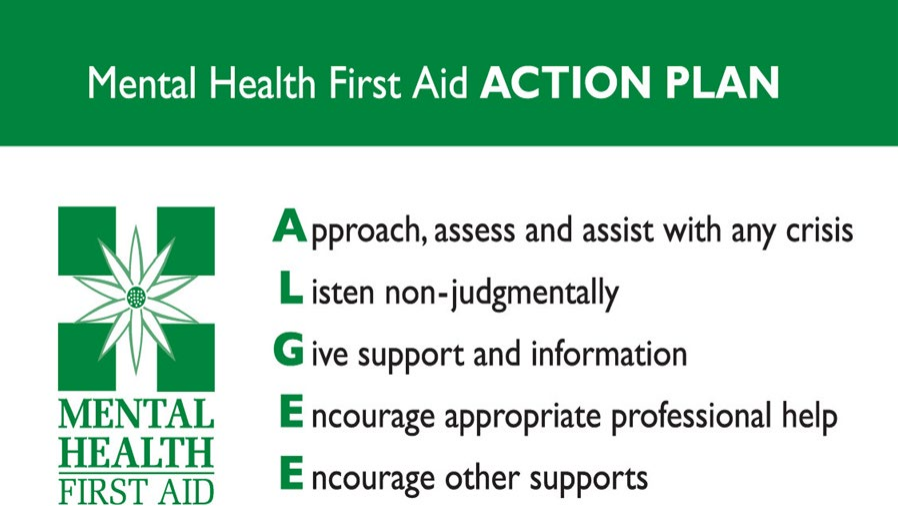
Mental Health First Aid Action Plan. MHFA Action Plan for providing Mental Health First Aid. Like the successful D.R.A.B.C Action Plan for emergency medical first aid, the MHFA Training and Research program uses the ALGEE Action Plan, to teach members of the public how to assist individuals with mental health problems to manage symptoms and seek appropriate help

Mental Health First Aid is ‘the help offered to a person developing a mental health problem, experiencing a worsening of an existing mental health problem or in a mental health crisis. The first aid is given until appropriate professional help is received or until the crisis resolves’ (p. 12) [27]. A Mental Health First Aid (MHFA) training program was developed in Australia in 2000 with the goal of improving recognition of mental disorders, reducing stigma and promoting appropriate help-seeking, self-care and support from others in the community [28]. The Standard MHFA course is delivered in a 12-h workshop and teaches the application of a Mental Health First Aid Action Plan to offer initial assistance to a person with a mental health problem or in a mental health crises [27] (Fig. 1).

It has since been evaluated in multiple studies in Australia and other countries, demonstrating its acceptability and effectiveness [29]. Remarkably, since 2001, the MHFA training program has been disseminated rapidly and gained recognition, with many countries licensing the MHFA course and adapting it to their specific cultural environment [28].

To increase the evidence base of the MHFA program, a series of Delphi studies have been conducted to develop expert consensus guidelines on what constitutes appropriate Mental Health First Aid for a range of mental health problems or crises [28], and to develop additional guidelines on provision of culturally-specific Mental Health First Aid for minorities in Australia [30, 31]. Recently, a consensus study was carried out to develop guidelines on important additional considerations when providing Mental Health First Aid to Iraqi refugees [32]. This evidence informed an adapted short 7-h Mental Health Literacy Course in how to assist Iraqi refugees with depression and PTSD problems or crises. This 7-h Mental Health Literacy Course was based on part of the MHFA training curriculum, with permission from Mental Health First Aid Australia. In the present study, this novel adapted training course was delivered to community-based workers and examined whether the training was effective in changing participants’ knowledge, increasing the quality of helping intentions and increasing positive behaviours and attitudes towards Iraqi refugees with depression and PTSD problems. It was hypothesized that the 1-day training intervention would be associated with an increase in participants’ knowledge about mental health problems, in the quality of helping intentions and behaviours and a decrease in participants’ negative attitudes towards Iraqi refugees with mental health problems.

## Methods

### Participants

Based on a power analysis, a desired sample size of n = 57 was required. This assumed that the training intervention would have a medium effect and made the conservative assumption that there was no correlation between pre- and post-training scores. With these assumptions this number would give 95% power to detect a medium effect size (d = 0.5) from pre- to post-training and follow-up with an alpha of 0.05.

A total of 86 participants were trained and responded the pre- and post-training questionnaires, with 45 (52%) completing the 6-month follow-up questionnaire.

Participants were community-based workers, based in Western Sydney, assisting Iraqi refugees on their resettlement. The training was advertised in major refugee health networks, by sending a flyer in an email format and a newsletter to several agencies that provide aid to humanitarian entrants.

Participants were volunteers who made contact with the principal investigator (MGU) for enrolment and other information. Workers were eligible to participate if they were working with refugee clients on a permanent basis and had no prior mental health training. Potential participants were excluded if they had university training (or degree) in mental health or had experience working in refugee mental health.

These workers were targeted for convenience sampling because they provide assistance to a group at very high risk of developing mental health problems [14]. Approval for this research was granted by the Western Sydney University Human Research Ethics Committee, reference number H11420.

### Intervention

The training intervention was a 7-h, classroom-style education program. It included didactic teaching using a PowerPoint presentation, films demonstrating how to provide assistance, small-group learning activities and whole-group discussion components. A teaching manual was developed to guide facilitation and ensure fidelity. Training was delivered by one primary instructor and principal investigator (MGU). The instructor undertook the standard Mental Health First Aid course in combination with one-on-one instructor session with MHFA Australia Founder Ms Betty Kitchener. The program content was presented across 2 sessions, morning and afternoon with two 15-min breaks and one longer lunchbreak. The units taught are presented in Table 1. The structure and content were developed based on the successful model devised by the MHFA program [27]. The information presented on symptoms, risk factors, warning signs, effective evidence-based treatments and early intervention was gleaned from reviews of current scientific literature [27]. The information provided on Mental Health First Aid strategies was based on existing MHFA guidelines for depression and traumatic events [33, 34]. In addition, the workshop included content based on the guidelines on important considerations when providing Mental Health First Aid to Iraqi refugees with depression and PTSD problems (details in Table 1) [32].

**Table 1:** Structure and content of the mental health literacy training course for workers assisting Iraqi refugees

Session one	Session two
Introductory activities	MHFA Action Plan^a^ for depression problems
Common mental illnesses occurring in the Australian population and Iraqi refugees^b^	Crisis first aid for suicidal thoughts and behaviours
Risk factors for mental health problems in Iraqi refugees^b^	Crisis first aid for non-suicidal self-injury MHFA Actions 2–5^a^
Iraq’s cultural diversity and Iraqi refugee intake in Australia^b^	Depression film^b^
Barriers and professional help-seeking and stigma associated with mental health problems in the Iraqi refugee community^b^	Anxiety Problems—PTSD in Iraqi refugees: Signs, symptoms and risk factors Interventions
Impact of mental illness	MHFA Action Plan for anxiety problems: Crisis first aid for panic attacks Crisis first aid after a traumatic event MHFA Actions 2–5^a^
The importance of early intervention and professional help	Culturally-adapted PTSD film^b^
The components of the MHFA Action Plan	Evaluation activity and conclusion
Cultural considerations and cross-cultural communication strategies when providing MHFA to Iraqi refugees^b^	
Depression: signs, symptoms, risk factors and effective interventions	

^a^Sourced from ALGEE—see Fig. 1 for details

^b^Sourced from important considerations when providing Mental Health First Aid to Iraqi refugees: a supplementary booklet for people working with Iraqi refugees

All participants were given a copy of the standard MHFA manual (3rd edition) [27] along with the booklet on important considerations when providing Mental Health First Aid to Iraqi Refugees [35].

To aid in the learning experience in the training session, the authors developed a culturally-sensitive video of a scripted drama depicting a male Iraqi refugee with mental health problems and a male teacher from an Adult Migrant English Program (AMEP) applying the Mental Health First Aid Action Plan.

### Measures

An assessment battery, including a range of self-report questionnaires (Additional file 1), was designed to measure change in knowledge, attitudes and the quality of helping intentions and behaviours in participants when providing first aid to Iraqi refugees. Information on the demographic characteristics of participants was also collected. Table 2 shows the variables measured across time points.

**Table 2:** Variables measured across time points

Variable measured	Pre-training	Post-training	6-month
Demographics	✓		
Recognition of PTSD	✓	✓	✓
Recognition of depression	✓	✓	✓
Knowledge of helpfulness of interventions for PTSD and depression	✓	✓	✓
Social distance towards PTSD and depression	✓	✓	✓
Stigma towards PSTD and depression			
1. Weak-not-sick subscale	✓	✓	✓
2. Dangerous/unpredictable subscale	✓	✓	✓
Offer help to a person with PTSD and depression related problems	✓	✓	✓
Confidence helping a person with PTSD and depression related problems	✓	✓	✓
Helping intentions	✓	✓	✓
General mental health knowledge	✓	✓	✓
Helping behaviours	✓		✓

### Recognition and knowledge of mental health problems

Recognition of mental health problems and knowledge of treatments was assessed using a culturally-valid mental health literacy survey developed by Slewa-Younan and collaborators [14]. The survey contained two vignettes. The first vignette described a fictional male Iraqi refugee, ‘Dawood’, who had been exposed to trauma prior to leaving Iraq and who was suffering symptoms of PTSD, according to criteria outlined in 5th edition of the Diagnostic and Statistical Manual of Mental Disorders (DSM 5) [36] (Additional file 2). The second vignette described a fictional female Iraqi refugee ‘Miriam’ who had been exposed to trauma prior to leaving Iraq and who was suffering symptoms of depression, according to criteria outlined in DSM 5 [36] (Additional file 2). Following the presentation of the vignettes, participants were asked in an open-ended format: `*What, if anything, do you think is wrong with* ‘*Dawood/Miriam*’*?.* The use of vignettes in mental health literacy research and mental health research more broadly, has been demonstrated to be ecologically valid [37, 38].

Coding for recognition of PTSD and depression was based on responses using key words. The labels were included in the ‘PTSD label category’ if they were very similar (or the same) to the correct label and contained any of the following wording: ‘*PTSD*’; ‘*post*-*traumatic stress disorder*’; ‘*post*-*trauma/tic stress/disorder*’ and ‘*PTS*’.

In the case of recognition of depression, labels included in this category were ‘*depression*’; ‘*depressed*’ and ‘*depressive*’. In terms of the ‘*general mental health problem’* category, labels that qualify to be included were ‘*mental problem’*, ‘*mental illness’* and ‘*mental disorder’*.

Participants’ beliefs about helpfulness of various interventions for the problems described in vignettes were also assessed. Specifically, participants were asked whether each of a number of interventions would be ‘Helpful’. For the analysis purposes, researchers treated helpfulness of interventions for PTSD and depression as continuous data. This procedure involved selecting interventions highly endorsed by health professionals (e.g. consulting a general practitioner or psychiatrist, undergoing treatment activities such as CBT or psychotherapy, and antidepressants) [39, 40]. Participants were awarded a point for each professional intervention they endorsed that was known to be effective as previously noted [39].

Knowledge of mental disorders was measured by a 15-item questionnaire specifically designed to cover information in the course. This was a modified version of a questionnaire previously utilised in MHFA evaluation trials [41]. The questionnaire included a mixture of statements reflecting both general knowledge of mental health problems in addition to culturally sensitive items required when assisting Iraqi refugees with mental health problems. Some examples of these items were ‘*Non*-*Western cultures tend to classify their distress using somatic or vegetative complaints such as headaches, disturbances of sleep or lack of energy*’; ‘*Antidepressant medications can be an effective treatment for most anxiety disorders*’ and ‘*PTSD and depressive disorders are the most prevalent mental illness in the Iraqi population’*. Response options for each item were ‘Agree’, ‘Disagree’ or ‘Don’t know’. Scoring was based on 1 point per correct response, providing a maximum score of 15.

### Attitudes

#### Social distance scale

Attitudes towards an Iraqi refugee with PTSD and depression were measured using a social distance scale developed by Link and colleagues [42]. Participants were asked the following items: *‘How you would feel about spending time with Dawood/Miriam?*’; ‘*Would you be happy: To move next door to Dawood/Miriam?; To spend some time socializing with Dawood/Miriam?; To make friends with Dawood/Miriam?; To work closely with Dawood/Miriam on a project at work?*; and; *To have Dawood/Miriam marry into your family?*’. Items were rated on a 4-point Likert scale composed of ‘1 = Yes, definitely’, ‘2 = Yes, probably’, ‘3 = Probably not’, and ‘4 = Definitely not’ as response options. In a large nationally representative sample of Australian adults, Yap et al. [37] found that social distance constituted a distinct factor of stigma that can be measured reliably (Cronbach’s alpha = 0.88). In the current study, the alphas were 0.91 and 0.92 for the PTSD and depression vignettes, respectively.

#### Personal stigma: weak-not-sick and dangerous/unpredictable subscales

The second measure used to assess negative attitudes of participants was the personal stigma in response to mental illness scale first devised by Griffiths and colleagues [43], and revised by Yap and colleagues [37]. This modified scale comprises two subscales. The first component is composed by 4 items which was labelled ‘weak-not-sick’ comprising personal stigma items about beliefs that the person was weak, not ill, could control their behaviour, and should be avoided. Some examples of this subcategory were ‘*Dawood/Miriam could snap out of it if they wanted*’ *or* ‘*Dawood’s/Miriam’s problem is a sign of personal weakness*’. A second component was named ‘Dangerous/unpredictable’ which is comprised by 5 items about dangerousness or unpredictability. Examples of the items of this category were ‘*Dawood/Miriam is dangerous*’ *or Miriam’s problem makes her unpredictable*’. Ratings for each item were made on a 5-point Likert scale in both subscales (‘1 = Strongly disagree’, ‘2 = Disagree’, ‘3 = Neither agree nor disagree’, ‘4 = Agree’, ‘5 = Strongly agree’). In this paper, the categories ‘Agree’ and ‘Strongly agree’ were combined and summed separately for each subscale.

The validity of these scales is shown by a factor analysis of personal stigma items from a large nationally representative sample of Australian adults, which found separate ‘weak-not-sick’ and ‘dangerous-unpredictable’ factors [37]. The scales were found to have reliabilities (Cronbach’s alpha) of 0.74 and 0.55 respectively. In the current study, the alphas for ‘weak-not-sick’ subscale were 0.77 and 0.73 for the PTSD and depression vignettes, respectively. Alphas for the ‘dangerous-unpredictable’ subscale were 0.62 and 0.75 for the PTSD and depression vignette, respectively.

Higher scores denoted higher levels of negative attitudes (social distance; weak-not-sick and Dangerous/unpredictable beliefs).

#### Intention to help

Intention to help an Iraqi refugee with mental health problems was measured using 2 questions devised in a previous MHFA evaluation study [41] administered at pre, post and follow-up time points. Firstly, participants were asked ‘*If Dawood/Miriam was one of your students or clients, I would help him/her*’. Rating for this item was made on a 7-point Likert scale (‘1 = Strongly disagree’, ‘2 = Mostly disagree’, ‘3 = Somewhat disagree’, ‘4 = Neither agree nor disagree’, ‘5 = Somewhat agree’,’ 6 = Mostly agree’, ‘7 = Strongly agree’).

Secondly, participants were asked to ‘*Describe all the things you would do to help Dawood/Miriam*’. De-identified and randomly intermixed responses were scored by an associated researcher (YM) who was blind to whether they were collected at pre-, post- or 6-month follow-up. A quality scoring system was utilised to measure the quality of these helping intentions devised by Yap and Jorm, [44]. This scoring system is based on the MHFA Action Plan taught in the third edition of the MHFA course [27]. Responses are awarded a point for each component of the Action Plan they mention (i.e. Approach the person, Assess and Assist with any crisis, Listen non-judgmentally, Give support and information, Encourage appropriate professional help and Encourage other supports) and an additional point per category where specific details are given (e.g. ‘Encourage the person to see a psychologist’ would receive two points for Encourage appropriate professional help). Responses can receive a minimum of 0 and a maximum of 2 points per component, giving a total score representing the quality of the response that ranges from 0 to 12. This score has previously been found to predict quality of subsequent helping behaviours, indicating its validity [45].

#### Confidence helping

To assess confidence, participants were asked ‘*How confident do you feel in helping someone with a problem like Dawood/Miriam?*’ extracted from a previous MHFA evaluation study [41]. Responses were rated on a 5-point Likert scale from ‘Not at all’ to ‘Extremely’. Confidence has been previously found to predict quality of subsequent helping behaviours [45], indicating the validity of such ratings.

#### Helping behaviours

In order to assess actual helping behaviours, participants were asked ‘*over the last 6* *months, has anyone that you have assisted (Iraqi refugee) had any sort of mental health problem?’ and ‘did you try to help the person with this problem?’* at pre-training and 6-month follow-up‘.

Participants were also asked to describe all the things they did to help the person (Iraqi refugee) retrospectively at pre-training and 6-month follow-up. Again, open ended responses rating were performed by an associated researcher (YM) based on the scoring system devised in a previous study [44].

Higher scores on these attitude measures denoted better quality of helping intentions and behaviours and, increased confidence when offering help.

### Design and analyses

The effectiveness of the training program was evaluated using an uncontrolled, repeated measures pre, post and six-month follow-up design.

Continuous variables are presented as means, whereas categorical variables are expressed as percentage (%) frequencies for pre-, post-training and follow-up points. All 86 participants completed the pre- and post-training questionnaire, however only 45 participants completed the 6-month follow-up questionnaire. All missing data were multiply imputed using multivariate normal imputation method assuming arbitrary missingness, as the missing value pattern showed a mixture of monotonic and non-monotonic missingness. The number of imputations performed was 10. Mixed-effects linear and logistic regression models were used to analyse continuous or binary outcome variables as appropriate.

The results for mixed effects regression based on multiply imputed datasets were finally pooled using Rubin’s method [46].

Finally, a paired sample t test was used to assess changes on actual helping behaviours, which was administered at two time points, pre-training and 6 months after the training completion.

An alpha level of 0.05 for all statistical tests was used. All analyses were performed using SAS (9.4) [47].

## Results

**Fig. 2:**
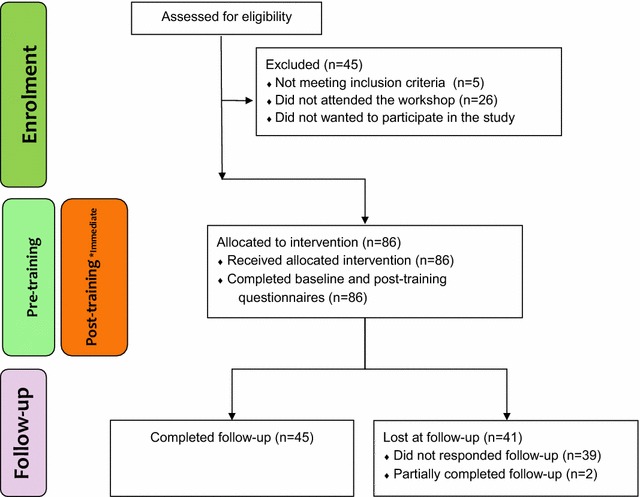
Study flowchart. *immediately after training

Training workshops were held between April and June 2016. Figure 2 displays participant flow through the research stages. Demographic data of the participants are presented in Table 3.

**Table 3:** Demographic characteristics of participants

Characteristics	Pre- and post-training (n = 86)	%	Follow-up (n = 45)	%
Gender				
Male	15	17.400	6	13.300
Female	71	82.600	39	86.700
Age in years, mean (SD)	44.1 (12.70)	–	45.79 (12.67)	
Qualifications				
Certificate	11	12.800	8	17.800
Diploma	13	15.100	7	15.600
Degree	45	52.300	20	44.400
Postgraduate	17	19.800	10	22.200
Employment Sector				
Government	33	38.400	23	51.100
Non-Government	53	61.600	22	48.900
Type of Role				
Community Worker Assisting Resettlement	28	32.600	7	15.600
English Tutor	26	30.200	16	35.600
High School Teacher	32	37.200	22	48.900
Country of Birth				
Australia	23	26.700	14	31.100
Middle East	21	24.400	11	24.400
Other	42	48.800	20	44.400

### Recognition and knowledge of PTSD and depression

Table 4 presents data on participants’ recognition and knowledge of and attitudes towards PTSD and depression across pre-, post-training and 6-month follow-up.

**Table 4:** Participants’ mental health literacy levels

Variables	Pre-training	Post-training	Follow-up	Cohen’s d for Pre versus post	OR for pre versus post	Cohen’s d for pre versus follow-up	OR for pre versus follow-up
Knowledge related to PTSD							
Problem recognised as ‘PTSD’ (%)^a^	56.900	76.500	75.1	–	3.540 *	–	3.220*
Problem recognised as ‘anxiety’ (%)^a^	15.600	33.700	18.8	–	2.940*	–	1.250
Problem recognised as a ‘general mental health problem’ (%)^a^	72.300	96.300	80.2	–	11.970***	–	1.730
Knowledge of helpfulness of interventions (mean, SD)	5.930 (2.140)	7.500 (1.730)	6.47 (2.23)	0.800***	–	0.240	–
Social Distance Scale (mean, SD)	10.050 (3.590)	9.330 (3.160)	8.56 (3.01)	0.210**	–	0.440***	–
Stigma (mean, SD):							
Weak-not-sick subscale	6.820 (.980)	5.990 (2.490)	5.95 (2.78)	0.430***	–	0.480	–
Dangerous/unpredictable subscale	11.220 (3.000)	9.730 (3.130)	9.99 (3.06)	0.480***	–	0.400	–
Offer help (mean, SD):	5.880 (1.780)	5.900 (2.020)	6.15 (1.84)	0.010	–	0.140	–
Confidence helping (mean, SD):	3.140 (1.230)	3.940 (0.940)	3.75 (1.05)	0.730***	–	0.530***	–
Helping intentions mean, SD)^b^	3.650 (1.620)	4.800 (2.260)	4.18 (1.78)	0.590***	–	0.310	–
Knowledge related to depression							
Problem recognised as ‘depression’ (%)^a^	28.400	39.50	45.100	–	1.730	–	2.080
Problem recognised as a ‘general mental health problem’ (%)^a^	79.700	98.80	95.600	–	18.990**	–	1.040
Knowledge of helpfulness of interventions (mean, SD)	8.170 (2.970)	10.79 (2.22)	9.310 (3.000)	0.990***	–	0.380**	–
Social Distance Scale (mean, SD)	9.360 (3.190)	9.32 (3.18)	8.220 (3.110)	0.010	–	0.360**	–
Stigma—depression vignette (mean, SD)							
Weak-not-sick subscale	6.690 (2.850)	6.14 (2.47)	6.220 (2.760)	0.200*	–	0.160	–
Dangerous/unpredictable subscale	9.480 (3.260)	9.220 (2.910)	9.460 (3.320)	0.080	–	0.000	–
Offer help (mean, SD):	5.880 (1.780)	5.900 (2.020)	6.150 (1.840)	0.010	–	0.140	–
Confidence helping (mean, SD)	3.370 (1.040)	4.110 (.770)	3.640 (.880)	0.800***	–	0.280**	–
Helping intentions (mean, SD)^b^	3.390 (1.590)	5 (2.360)	3.470 (1.910)	0.800***	–	0.040	–
General mental health knowledge							
Mental Health First Aid knowledge quiz (mean, SD)	10.020 (2.200)	12.030 (2.050)	8.980 (2.110)	0.940***	–	− 0.840 ***	–
Helping behaviors towards an Iraqi refugees with mental health problems	(n = 16)		(n = 16)				
ALGEE total score (mean, SD)^b^	3.250 (1.160)	–	3.450 (1.950)	–	–	0.120	–

^a^Multiple responses were permitted

^b^Approach the person, assess and assist with any crisis, listen non-judgmentally, give support and information, encourage appropriate professional help and encourage other supports

* P < 0.05, ** p < 0.01, *** p < 0.001

In response to the question ‘What, if anything, do you think is wrong with ‘Dawood’?, 56% of participants correctly recognised the problem as ‘PTSD’ before training and 77% of them did after training (*p* = 0.001). This improved recognition of PTSD was retained at follow-up, with 82% of participants correctly noting the disorder in the vignette (*p* = 0.032).

To assess whether recognition of the problem in the PTSD vignette as just a ‘general mental health problem’ improved over time, the frequencies of all other responses representing a mental health related label (‘mental illness’, ‘mental problem’, ‘mental breakdown’, ‘mental issue’) were included. There was an increase of problem recognition from pre- to post-training (*p* < 0.001), with percentages increasing from 71 to 96.5%, and remaining high at follow-up, with 98% of participants recognising the problem in the vignette as a mental health problem.

In response to the depression vignette, 69% of participants were able to correctly identify Miriam’s problem as ‘depression’ at pre-training. Immediately following training, this increased to 83.5% at post-training and then to 82.2% at follow-up. However, these gains between pre- to post-training and follow-up rates were not significant. When analysing the number of participants recognising the problem described in the depression vignette as a ‘general mental health problem’, there was a significant increase from 77.9% at pre-training to 97.7% at post-training (*p* = 0.004). Recognition remained very high at follow-up, with 95.6% participant noting the vignette as a general mental health problem (*p* = 0.921).

A significant improvement in participants’ knowledge about mental health problems and Mental Health First Aid knowledge was noted from pre- to post-training (*p* < 0.001). However, there was a significant reduction of knowledge at 6-month follow-up (*p* < 0.001).

### Beliefs of helpfulness of treatments for PTSD and depression

Following training, participants were more likely to endorse professional recognised treatment for PTSD (*p* < 0.001). However, these gains were not maintained at follow-up. In addition, a significant increase was noted for the endorsement of professional interventions for depression after training (*p* < 0.001) and at follow-up (*p* = 0.010).

### Attitudes towards PSTD and depression

A reduction in levels of personal stigma associated with the PTSD vignette was found post-training, for both the subscales of ‘weak-not-sick’ (*p* < 0.001) and ‘dangerous/unpredictable beliefs’ (*p* < 0.001). Scores remained lower at follow-up compared with pre-training, however the difference was not significant.

Participants’ social distance scale scores decreased substantially (*p* = 0.006) after training with regards to PTSD vignette. A significant decrease in social distance towards PTSD from pre-training to follow-up was also found (*p* < 0.001).

When considering the depression vignette, the personal stigma subscale of ‘weak-not-sick’ decreased after training (*p* = 0.013) and remained lower at follow-up, although this decrease was not significant.

In terms of social distance towards depression, participants’ scores decreased significantly from pre-training to follow-up (*p* = .007).

### Confidence in providing help

Confidence helping the person in the PTSD vignette (*p* < 0.001 *at post*-*training, p* < 0.001 *at follow*-*up*) increased, as did confidence helping the person in the depression vignette (*p* < 0.001 *at post training, p* = 0.003 *at follow*-*up*).

### Intention to provide help

Helping intentions scores for the PTSD and depression vignettes were only significantly different between pre-training and post-training (*p* < 0.001). Greater quality of helping intentions regarding the depression vignette were also reported at post-training (*p* < 0.001).

### Helping behaviours

A total of 16 participants provided responses to indicate that they had in fact actually tried to help someone and described in detail these interventions that allowed for measure of helping behaviours to be scored, with a comparison undertaken between pre-training and follow-up time points. Although not statistically significant, there was a slight increase in scores between the two time points.

## Discussion

The current study sought to evaluate whether the tailored Mental Health Literacy Course on providing help to Iraqi refugees with mental health problems was effective in changing participants’ knowledge, intentions, confidence and attitudes. Post intervention, our results demonstrated a significant impact on most of the mental health literacy measures, namely improving recognition of PTSD and depression, increasing knowledge of mental health problems and evidence-based interventions, improving participants’ intention to help and decreasing negative attitudes. Of note was the significant improvement in participant’s confidence in helping an Iraqi refugee, which was found at both post intervention and at 6 month follow-up.

Recognising when an Iraqi client has symptoms of PTSD or depression is an important skill to have when workers are tasked with providing services to assist in their resettlement, such as English tuition. Our training provided a learning platform that assisted participants through the provision of culturally-appropriate resources developed to improve understanding around the Iraqi culture (e.g. Iraqi patriarchal society, males rarely speaking out, and communication barriers) [32]. This may have contributed to the high proportion of participants accurately identifying the problem presented in the vignette even at 6 months following training. Further, this adapted Mental Health Literacy Course included best practice guidelines on how to help Iraqi refugees with mental health problems, and a culturally specific film that provided strategies on how to provide initial help to an Iraqi refugee with PTSD-related problems, which again may have contributed to the significant improvement in most mental health literacy measures.

Previous Australian based research has demonstrated the existence of dual preferences for treatments amongst resettled Iraqi refugees, such as seeking help from a psychiatrist in addition to reading religious texts [14]. This information was discussed in this tailored program in order to provide participants with a clearer cultural understanding. However, in order to prepare the first aider so that they can encourage a person to get appropriate professional help and other supports, information on evidence based interventions (psychological, medical and self-help strategies) were provided as part of the training program [48, 49]. Our training was effective in increasing knowledge on evidence-based interventions, such as consulting a general practitioner or psychiatrist, undergoing treatment activities such as CBT or psychotherapy, and antidepressants.

Additionally, other positive and significant impacts of this training were found on levels of confidence in helping an Iraqi refugee with PTSD and depression-related problems. Confidence levels increased markedly after training and were maintained at follow-up. We believe this could be associated with the ability to recognise the problem as ‘PTSD’ or ‘depression’ in a cross-cultural setting, as well as the increased level of knowledge about professionally-aligned helpful interventions for managing these conditions. Further, a significant change was noted in social distance scores regarding PTSD, which reduced over time. It could be argued that this change in the attitudes of participants towards someone experiencing PTSD-related problems can be linked again, to their improved capacity to recognise the problem as a genuine health problem.

However, not all the mental health literacy components demonstrated improvement. It is difficult to postulate the reasons for this and in future incorporating qualitative interviews of participants may assist in teasing out such findings. It is important to note that this was a pilot project to assess the usefulness of training informed by the guidelines on important considerations when providing Mental Health First Aid to Iraqi refugees in Australia. However, for broader dissemination this short 7-h training course may be best integrated into the Standard 12-h MHFA course, which has been widely disseminated in Australia and in over 20 other countries.

Several limitations of this study must be noted. This study utilised an uncontrolled design with a small sample size. As the current study formed part of a PhD project, limited funding and timeframe required a more pragmatic approach to be adopted. An intervention with a randomised control group design using a larger sample size would be ideal. In addition, this study used a convenience sample of volunteers and thus unlikely to be representative to all community-based workers. Other methodological limitations include the self-reported nature of the assessments and that the scoring of helping intentions was undertaken by only one blinded judge preventing an assessment of inter-rater reliability.

Further, there was no evaluation as to the impact of the training of these workers on the actual mental health or help-seeking of their Iraqi refugee clients. In addition, there was a large dropout rate of participants at follow-up, although it was dealt with using multiple imputation. Future studies should use more assertive follow-up in order to reduce the drop-out rate. Other future directions should consider efforts directed at improving the mental health literacy of the Iraqi refugees themselves, as such programs would complement the current study as part of a series of efforts to improve the mental health outcomes of resettling refugees.

Strengths of this study include being the first program of its kind that seeks to train community-based workers on how to provide culturally-appropriate initial help to individuals from an Iraqi refugee background with mental health problems. While there has been an increased emphasis on cultural competency in mental health care and the delivery of evidence-based psychosocial services for ethnic groups [23, 50], to date, culturally-appropriate psychoeducation initiatives at a community based level are rare. Secondly, by utilising content from the highly-successful standard MHFA training curriculum and supplementing it with culturally-appropriate guidelines developed through an expert panel Delphi study [32], a culturally-salient training program with appropriate teaching resources was developed. Finally, the processes and mechanism utilised in this study can potentially serve as a framework on how to develop culturally-appropriate psychoeducation to workers assisting Iraqi or other refugee and culturally-and-linguistically-diverse groups in Australia. Further, with appropriate modifications to the resource booklet [35] accompanying this training, this could be used in other developed English speaking countries. However, the effects of the training in other countries would need evaluation.

## Conclusion

This research demonstrates that the tailored program was effective in improving recognition of PTSD and depression, reducing negative attitudes towards PTSD and depression problems, changing beliefs regarding treatment to align with those of mental health professionals, and improving confidence when helping an Iraqi refugee with PSTD and depression problems. To the best of our knowledge, this is the first time that a psychoeducation program has been adapted for workers to assist a refugee group. It is a recommendable way to improve and better equip staff on how to respond to mental health crises and offer Mental Health First Aid in a culturally-aware way to Iraqi refugees and is a necessary part of the ongoing efforts needed to improve the resettlement outcomes of refugees.

## Additional files

**Additional file 1.** Assesment Battery.

**Additional file 2.** PTSD Vignette and Depression Vignette.

## Abbreviations

NSW: New South Wales
MHL: mental health literacy
PTSD: posttraumatic stress disorder
MHFA: Mental Health First Aid
AMEP: Adult Migrant English Program
CBT: Cognitive behavioural therapy
WHO: World Health Organisation
