# Supplementary material for: Improving the capacity of community-based workers in Australia to provide initial assistance to Iraqi refugees with mental health problems: an uncontrolled evaluation of a Mental Health Literacy Course
Source: Int J Ment Health Syst. 2018 Jan 15;12:2. doi: 10.1186/s13033-018-0180-8 (PMC5769322; doi:10.1186/s13033-018-0180-8)
Supplement: Supplementary file 1 — Additional file 1. Assesment Battery. [file 13033_2018_180_MOESM1_ESM.pdf]

## Recognition of Problems in Vignettes, Intentions to Help and Confidence in Helping

**Below is a story regarding a hypothetical person called Dawood. The box below describes how he has been recently.**

**<u>Vignette 1</u>**

***Dawood is a 37 year old married man with 3 children, a daughter aged 7, and two sons, aged 5 and 3. Dawood has been living in Australia for the past year and has attended his local GP on several occasions with the primary complaint of an inability to sleep. The problem with his sleep started just before he left his homeland, Iraq, four years ago. Prior to leaving Iraq, Dawood was kidnapped by insurgents and held captive until his brother paid the demanded ransom. During his kidnapping, Dawood was beaten and threatened with death. He reports that during the kidnapping he felt intense fear and helpless. He reports constant nightmares in which images of death, killing and being kidnapped by masked men disturb his sleep. He avoids talking about his attack and watching Iraqi news channels in case there is a story about people being kidnapped and murdered. He is easily startled when he hears loud sounds such as a car backfiring or fireworks. He has very little interest in things around him, including his children's lives and feels little affection towards them. Finally, when questioned on how he views his future and plans for his life, Dawood replies that he does not have a future and doesn't believe he will live a long life.***

* 1. What, if anything, do you think is wrong with Dawood?

* 2. If Dawood was one of your students or clients, I would help him.

| Strongly disagree | Mostly disagree | Somewhat disagree | Neither agree nor disagree | Somewhat agree | Mostly agree | Strongly agree |
|---|---|---|---|---|---|---|
| ○ | ○ | ○ | ○ | ○ | ○ | ○ |

* 3. Describe all the things you would do to help Dawood.

* 4. How confident do you feel in helping someone with a problem like Dawood’s?

| Not at all | A little bit | Moderately | Quite a bit | Extremely |
|---|---|---|---|---|
| ○ | ○ | ○ | ○ | ○ |

* 5. If Dawood were to seek help for his problem from any of the following, is it likely to be helpful, harmful or neither for him? (Select one response for each line)

| | Helpful | Neither | Harmful | Depends | Do not know |
|---|---|---|---|---|---|
| A typical family GP or doctor | ○ | ○ | ○ | ○ | ○ |
| A psychiatrist | ○ | ○ | ○ | ○ | ○ |
| A psychologist | ○ | ○ | ○ | ○ | ○ |
| Dawood tries to deal with his problems on his own | ○ | ○ | ○ | ○ | ○ |
| Vitamins and minerals, tonics or herbal medicines | ○ | ○ | ○ | ○ | ○ |
| Antidepressants | ○ | ○ | ○ | ○ | ○ |
| Reading the Bible or Koran | ○ | ○ | ○ | ○ | ○ |
| Becoming more physically active | ○ | ○ | ○ | ○ | ○ |
| Reading about people with similar problems and how they have dealt with them | ○ | ○ | ○ | ○ | ○ |
| Getting out and about more | ○ | ○ | ○ | ○ | ○ |
| Courses on relaxation, stress management, meditation or yoga | ○ | ○ | ○ | ○ | ○ |
| Cutting out alcohol altogether | ○ | ○ | ○ | ○ | ○ |
| Psychotherapy | ○ | ○ | ○ | ○ | ○ |
| Cognitive behaviour therapy | ○ | ○ | ○ | ○ | ○ |
| A special diet or avoiding certain foods | ○ | ○ | ○ | ○ | ○ |
| Consulting a book that gives information about his health problem | ○ | ○ | ○ | ○ | ○ |

| | Helpful | Neither | Harmful | Depends | Do not know |
|---|---|---|---|---|---|
| Receiving information about his problem from a health educator | ○ | ○ | ○ | ○ | ○ |
| Visiting a Religious leader such as a priest or Imam | ○ | ○ | ○ | ○ | ○ |

* 6. The next few questions contain statements about Dawood's problem. Please indicate how strongly YOU PERSONALLY agree or disagree with each statement.

| | Strongly disagree | Disagree | Neither agree nor disagree | Agree | Strongly agree |
|---|---|---|---|---|---|
| a. Dawood could snap out of it if he wanted | ○ | ○ | ○ | ○ | ○ |
| b. Dawood's problem is a sign of personal weakness | ○ | ○ | ○ | ○ | ○ |
| c. Dawood's problem is not a real medical illness | ○ | ○ | ○ | ○ | ○ |
| d. Dawood is dangerous | ○ | ○ | ○ | ○ | ○ |
| e. It is best to avoid Dawood so that you don't develop this problem yourself | ○ | ○ | ○ | ○ | ○ |
| f. Dawood's problem makes him unpredictable | ○ | ○ | ○ | ○ | ○ |
| g. You would not tell anyone if you had a problem like Dawood's | ○ | ○ | ○ | ○ | ○ |
| h. I would not employ someone if I knew they had a problem like Dawood's | ○ | ○ | ○ | ○ | ○ |
| i. I would not vote for a politician if I knew they had suffered a problem like Dawood's | ○ | ○ | ○ | ○ | ○ |

* 7. The following questions ask how you would feel about spending time with Dawood. Would you be happy:

| | Yes, definitely | Yes, probably | Probably not | Definitely not |
| --- | --- | --- | --- | --- |
| a. To move next door to Dawood? | ◯ | ◯ | ◯ | ◯ |
| b. To spend an evening socializing with Dawood? | ◯ | ◯ | ◯ | ◯ |
| c. To make friends with Dawood? | ◯ | ◯ | ◯ | ◯ |
| d. To work closely with Dawood on a project at work? | ◯ | ◯ | ◯ | ◯ |
| e. To have Dawood marry into your family? | ◯ | ◯ | ◯ | ◯ |

## Recognition of Problems in Vignettes, Intentions to Help and Confidence in Helping

**Below is a story regarding a hypothetical person called Miriam. The box below describes how she has been recently.**

**Vignette 2**

**Miriam is a 37 year old married woman with 2 children, a daughter aged 7, and son aged 5. Miriam migrated to Australia as a refugee after her father was killed in Iraq almost 3 years ago. Miriam has been living in Australia for the past year and has attended her local GP on several occasions with the following complaints; she feels sad and cries all the time and has little energy or motivation to do daily activities. Additionally she reports a loss of appetite, which has resulted in a weight loss of 5 kilos. On further query from her doctor, Miriam reports feelings of worthlessness and immense guilty because she cannot help her uncles and cousins back home in Iraq.**

* 8. What, if anything, do you think is wrong with Miriam?

* 9. If Miriam was one of your students or clients, I would help her.

| Strongly disagree | Mostly disagree | Somewhat disagree | Neither agree nor disagree | Somewhat agree | Mostly agree | Strongly agree |
|---|---|---|---|---|---|---|
| ○ | ○ | ○ | ○ | ○ | ○ | ○ |

* 10. Describe all the things you would do to help Miriam.

* 11. How confident do you feel in helping someone with a problem like Miriam's?

| Not at all | A little bit | Moderately | Quite a bit | Extremely |
|---|---|---|---|---|
| ○ | ○ | ○ | ○ | ○ |

* 12. If Miriam were to seek help for her problem from any of the following, is it likely to be helpful, harmful or neither for her? (Select one response for each line)

| | Helpful | Neither | Harmful | Depends | Do not know |
|---|---|---|---|---|---|
| A typical family GP or doctor | ○ | ○ | ○ | ○ | ○ |
| A psychiatrist | ○ | ○ | ○ | ○ | ○ |
| A psychologist | ○ | ○ | ○ | ○ | ○ |

| | Helpful | Neither | Harmful | Depends | Do not know |
|---|---|---|---|---|---|
| Miriam tries to deal with her problems on her own | | | | | |
| Vitamins and minerals, tonics or herbal medicines | | | | | |
| Antidepressants | | | | | |
| Reading the Bible or Koran | | | | | |
| Becoming more physically active | | | | | |
| Reading about people with similar problems and how they have dealt with them | | | | | |
| Getting out and about more | | | | | |
| Courses on relaxation, stress management, meditation or yoga | | | | | |
| Cutting out alcohol altogether | | | | | |
| Psychotherapy | | | | | |
| Cognitive behaviour therapy | | | | | |
| A special diet or avoiding certain foods | | | | | |
| Consulting a book that gives information about her health problem | | | | | |
| Receiving information about her problem from a health educator | | | | | |
| Visiting a Religious leader such as a priest or Imam | | | | | |

* 13. The next few questions contain statements about Miriam’s problem. Please indicate how strongly YOU PERSONALLY agree or disagree with each statement.

| | Strongly disagree | Disagree | Neither agree nor disagree | Agree | Strongly agree |
|---|---|---|---|---|---|
| a. Miriam could snap out of it if he wanted | ○ | ○ | ○ | ○ | ○ |
| b. Miriam’s problem is a sign of personal weakness | ○ | ○ | ○ | ○ | ○ |
| c. Miriam’s problem is not a real medical illness | ○ | ○ | ○ | ○ | ○ |
| d. Miriam is dangerous | ○ | ○ | ○ | ○ | ○ |
| e. It is best to avoid Miriam so that you don’t develop this problem yourself | ○ | ○ | ○ | ○ | ○ |
| f. Miriam’s problem makes her unpredictable | ○ | ○ | ○ | ○ | ○ |
| g. You would not tell anyone if you had a problem like Miriam’s | ○ | ○ | ○ | ○ | ○ |
| h. I would not employ someone if I knew they had a problem like Miriam’s | ○ | ○ | ○ | ○ | ○ |
| i. I would not vote for a politician if I knew they had suffered a problem like Miriam’s | ○ | ○ | ○ | ○ | ○ |

* 14. The following questions ask how you would feel about spending time with Miriam. Would you be happy:

| | Yes, definitely | Yes, probably | Probably not | Definitely not |
|---|---|---|---|---|
| a. To move next door to Miriam? | ◯ | ◯ | ◯ | ◯ |
| b. To spend an evening socializing with Miriam? | ◯ | ◯ | ◯ | ◯ |
| c. To make friends with Miriam? | ◯ | ◯ | ◯ | ◯ |
| d. To work closely with Miriam on a project at work? | ◯ | ◯ | ◯ | ◯ |
| e. To have Miriam marry into your family? | ◯ | ◯ | ◯ | ◯ |

## Mental health first aid knowledge

* 15. Please indicate whether you agree or disagree with each statement, or if you don’t know.

| | Disagree | Agree |
|---|---|---|
| In helping an Iraqi refugee with mental health problems, it is useful to understand the historical context of the conflicts and wars that Iraq has been engaged in over the past 30 years, which may affect the person's mental health and help-seeking behaviour. | ○ | ○ |
| Half of all people who experience a mental illness have their first episode by age 18. | ○ | ○ |
| PTSD and Depressive disorders are the most prevalent mental illness in the Iraqi population. | ○ | ○ |
| Ongoing conflicts in Iraq might worsen or trigger mental health problems in the person. | ○ | ○ |
| If a person who is depressed does not want to seek professional help, it is important to force them to if you can. | ○ | ○ |
| When communicating using a raised voice with a person with limited English comprehension is helpful. | ○ | ○ |
| Iraqis tend to talk openly about their mental health problems. | ○ | ○ |
| Exercise can help relieve depression. | ○ | ○ |

| | Disagree | Agree |
|---|---|---|
| Recovery from anxiety disorders requires facing situations which are anxiety provoking | ○ | ○ |
| Antidepressant medications can be an effective treatment for most anxiety disorders. | ○ | ○ |
| Non-Western cultures tend to classify their distress using somatic or vegetative complains such as headaches, disturbances of sleep or lack of energy. | ○ | ○ |
| People with mental illnesses are much more likely to be smokers. | ○ | ○ |
| It is not a good idea to ask someone if they are feeling suicidal in case you put the idea in their head. | ○ | ○ |
| It is best to get someone having a panic attack to breathe into a paper bag. | ○ | ○ |
| It is very important to offer a professional interpreter to the person, where one is needed. | ○ | ○ |

**Self-reported supportive behaviours if someone from an Iraqi refugee background develops a mental health problem**

* 16. Over the last 6 months, has anyone that you assist (Iraqis) had any sort of mental health problem? (a “mental health problem”, is a cluster of symptoms that affects a person’s thinking, emotional state and behaviour, and disrupts the person’s ability to work or carry out other daily activities and engage in satisfying personal relationships. The problem lasts for a period of weeks or more).

| Yes | No | Don't know | Refused |
|---|---|---|---|
| ○ | ○ | ○ | ○ |

* 17. Over the last 6 months, did you try to help the person with this problem?

| Yes (Go to 29) | No (Go to 28) |
|---|---|
| ○ | ○ |

* 18. Describe all the things you did to help.
