# Supplementary material for: Improving the capacity of community-based workers in Australia to provide initial assistance to Iraqi refugees with mental health problems: an uncontrolled evaluation of a Mental Health Literacy Course
Source: Int J Ment Health Syst. 2018 Jan 15;12:2. doi: 10.1186/s13033-018-0180-8 (PMC5769322; doi:10.1186/s13033-018-0180-8)
Supplement: Supplementary file 2 — Additional file 2. PTSD Vignette and Depression Vignette. [file 13033_2018_180_MOESM2_ESM.doc]

**Additional files 2**

**Additional file 3 – Depression Vignette**

**Miriam is a 37 year old married woman with 2 children, a daughter aged 7, and son aged 5. Miriam migrated to Australia as a refugee after her father was killed in Iraq almost 3 years ago. Miriam has been living in Australia for the past year and has attended her local GP on several occasions with the following complaints; she feels sad and cries all the time and has little energy or motivation to do daily activities. Additionally she reports a loss of appetite, which has resulted in a weight loss of 5 kilos. On further query from her doctor, Miriam reports feelings of worthlessness and immense guilty because she cannot help her uncles and cousins back home in Iraq.**

**Additional file 2 – PTSD Vignette**

**Dawood is a 37 year old married man with 3 children, a daughter aged 7, and two sons, aged 5 and 3. Dawood has been living in Australia for the past year and has attended his local GP on several occasions with the primary complaint of an inability to sleep. The problem with his sleep started just before he left his homeland, Iraq, four years ago. Prior to leaving Iraq, Dawood was kidnapped by insurgents and held captive until his brother paid the demanded ransom. During his kidnapping, Dawood was beaten and threatened with death. He reports that during the kidnapping he felt intense fear and helpless. He reports constant nightmares in which images of death, killing and being kidnapped by masked men disturb his sleep. He avoids talking about his attack and watching Iraqi news channels in case there is a story about people being kidnapped and murdered. He is easily startled when he hears loud sounds such as a car backfiring or fireworks. He has very little interest in things around him, including his children’s lives and feels little affection towards them. Finally, when questioned on how he views his future and plans for his life, Dawood replies that he does not have a future and doesn’t believe he will live a long life.**
